# miRNAs in Prediction of Prognosis in Clear Cell Renal Cell Carcinoma

**DOI:** 10.1155/2017/4832931

**Published:** 2017-12-17

**Authors:** LongJiao Ran, Jian Liang, Xin Deng, JinYu Wu

**Affiliations:** ^1^Ruikang Hospital Affiliated to Guangxi University of Chinese Medicine, 10 East China Road, Nanning, Guangxi Zhuang Autonomous Region, China; ^2^The First Affiliated Hospital of Guangxi University of Chinese Medicine, Nanning, China

## Abstract

Renal cell carcinoma (RCC) is the most common type of urinary malignancy. Clear cell renal cell carcinoma (ccRCC) is the predominant RCC subtype, accounting for 70–80% of RCC. In recent years, miRNAs have been found to be closely associated with the outcome of the patients with ccRCC. In this review, we summarize recent advances in research exploring the role of miRNAs in predicting prognosis in patients with ccRCC.

## 1. Introduction

Renal cell carcinomas (RCC) are malignancies derived from renal tubular epithelial cells, of which clear cell renal cell carcinoma (ccRCC) is the predominant pathological subtype. Since ccRCC is not sensitive to traditional radiotherapy or chemotherapy, and mRCC targeted therapies are usually expensive, ccRCC is most often treated with radical or partial nephrectomy [1]. In the early stage of the disease, ccRCC does not cause specific signs or symptoms, and approximately one-third of patients have distant metastases at the time of diagnosis [2]. Although surgical resection can effectively resolve ccRCC, 20 to 40% of patients still develop local recurrence or distant metastasis [3, 4] after surgery.

MicroRNAs (miRNAs) are small noncoding RNAs of 20–22 nucleotides. In ccRCC tissues, aberrant expression of some miRNAs has been observed [5, 6]. Measuring the expression of these miRNAs can distinguish ccRCC from normal tissue [7, 8], predict prognosis [9, 10], and highlight potential therapeutic targets [11, 12]. A previous study characterized an miRNA signature of 22 miRNAs that, among 147 miRNA profiles from 411 ccRCC patients, was independently correlated with ccRCC outcomes [13]. Recently miRNAs have attracted more and more attention due to their special associations with the prognosis of ccRCC. In this review, we discuss the involvement of miRNAs in ccRCC in the aspects, namely, pathologic grade or stage, recurrence and metastasis, and survival.

## 2. Pathological Grade or Stage

Preoperative prediction of the pathological grade or stage can facilitate the formulation and implementation of individualized treatment plan. In recent years, miRNAs have been found to be associated with pathological grade and stage. Ishihara et al. found remarkably low levels of miR-23B and miR-27B in ccRCC tissues which are to be associated with advanced pathological stage (defined as stage 3, *P* = 0.024) and advanced grade (grade 3, *P* = 0.0233) of ccRCC [14]. Abnormal expression of miR-23B and miR-27B was correlated with unfavorable overall survival, and restoration of these abnormalities resulted in inhibition of ccRCC growth and metastasis. The miR-23B/27B cluster was thus an accurate prognostic marker of pathological outcomes in ccRCC. Upregulation of miR-29b has been found in both malignant renal tissue and* in vitro* cultured RCC cell lines [15]. Elevation in miR-29b expression was associated with unfavorable clinical stage (*P* = 0.026) and overall survival (*P* = 0.009) in patients with ccRCC, whereas miR-29b accelerated the growth and migration of RCC cells through transcriptional regulation of KIF1B.

A 17.5-fold elevation in miR-125b expression was found to be correlated with a higher Fuhrman grade (*P* < 0.05) and advanced tumor-node-metastasis (TNM) stage (III + IV versus I + II, *P* = 0.131) in 276 cases of ccRCC [16]. Multivariate Cox analysis demonstrated miR-125b to be an independent prognostic factor (HR 1.860, 95% CI 1.059–3.269, *P* = 0.030) of ccRCC. Furthermore, the combination of miR-125b and TNM stage (*C*-index 0.715, 95% CI 0.656–0.773) improved the ccRCC prognosis predictive accuracy of TNM alone (*C*-index 0.664, 95% CI 0.614–0.715), reflecting the synergistic predictive ability of the two parameters.

Taken together, this evidence may provide important theoretical support for the prediction and evaluation of ccRCC prognosis. MiRNAs alone or particularly in combination with other clinicopathological features represent valuable tools for prognostic stratification of RCC patients. However, the possible mechanisms by which these miRNAs are involved in the development and progression of ccRCC remain largely unknown. These unsolved issues may be overcome by optimization of renal resection surgery and improved detection of various miRNAs in the future.

## 3. Recurrence and Metastasis

Analysis of 111 RCC specimens revealed a significantly higher degree of methylation in mir-124-3 CpG islands than normal tissues, which was associated with the occurrence of distant metastasis (*P* < 0.0001), high pathological grade (*P* = 0.0063), and increased risk of disease recurrence (*P* = 0.0005) [17]. These data suggested miR-124-3 as an optimal candidate biomarker for risk stratification of RCC patients. Also, high levels of miR-27a-3p have been shown to be involved in RCC progression [18]. In a multivariate Cox proportion hazard model, high miR-27a-3p expression conferred a 2.71-fold increased risk of ccRCC recurrence (HR 2.71, 95% CI 1.23–6.42, *P* = 0.0131), indicating miR-27a-3p as an independent prognostic factor to predict ccRCC recurrence. In the study conducted by Huang et al. [19], patients with hematogenous metastatic ccRCC had lower levels of miR-30a, higher tumor microvessel density, and higher levels of DLL4 expression than those without metastasis or with only lymphatic metastasis. The 3-year follow-up data from 65 ccRCC patients without synchronous metastases demonstrated that patients with high levels of miR-30a exhibited a lower probability of hematogenous spread and longer metastasis-free survival than those with low miR-30a levels. Thus, miR-30a is independently predictive of ccRCC hematogenous metastasis, which can facilitate individualized therapy to reduce metastasis-specific mortality. Similarly, miR-646 expression decreased with the ccRCC progression from nonmetastasis or lymphatic metastasis to distant metastasis [20]. Five-year follow-up data from 70 ccRCC patients without metastases showed that ccRCC patients with high levels of miR-646 achieved better metastasis-free survival time (*P* = 0.012). Reduced miR-646 expression was identified as an independent predictor of ccRCC distant metastasis. The underlying mechanism might involve mitogen-activated protein kinase (MAPK) pathway by targeting nin one binding protein (NOB1), as evidenced by a concomitant, inverse change in NOB1 level. Another miRNA identified to be associated with ccRCC metastasis is miR-30c [21]. miR-30c was found to be downregulated in both primary and metastatic lesions. In addition, overexpression of miR-30c decreased the migration and invasion capacity of* in vitro* cultured ccRCC cells. These data suggest that alterations in miR-30c expression predict early distant metastases of ccRCC, and restoration of its levels helps to reduce the invasiveness of ccRCC.

Fu et al. found high miR-125b levels to be correlated with poor survival rate (*P* = 0.007) and shorter recurrence-free survival (*P* = 0.002) among 276 ccRCC patients undergoing nephrectomy [16]. Overall, ccRCC patients with high miR-125b levels were at high risk of ccRCC recurrence (HR 2.396, 95% CI 1.365–4.778, *P* = 0.005), but stratification by clinical stage revealed that this marker was only predictive in those at an advanced clinical stage (T2–4, HR 6.366, 95% CI 2.754–25.508, *P* = 0.001) and not in those at an early stage (T1, HR 1.507, 95% CI 0.614–3.857, *P* = 0.363). These data again confirm the significance of miRNA and their alterations in early prediction of recurrence and survival of patients with ccRCC after nephrectomy. MiR-122 and miR-514 have also been validated as differentially expressed miRNA markers in ccRCC recurrence after radical nephrectomy [22]. Expression of miR-514 is significantly downregulated while miR-122 is upregulated in primary and metastatic lesions. However, only miR-514 remained as an independent factor predicting tumor recurrence in the final Cox regression model.

Taken together, miRNAs play a role in predicting the occurrence of ccRCC and postoperative recurrence and metastasis. Thus, miRNAs are useful tools facilitating personalized therapy selection and individualized follow-up schedule. However, current evidence is sparse and largely comes from limited number of events or* in vitro* experiments. The precise role of miRNAs in ccRCC, and specific molecular mechanisms involved, deserves further investigation. Such research may allow survival and quality of life of such patients to be improved.

## 4. Survival

Abnormalities in miRNA, upregulation or downregulation, predict overall survival (OR) or disease-free survival (DFS). It has been reported that miR-194 expression decreases gradually as normal renal tissues develop into primary ccRCC and is further decreased in metastatic lesions [10]. Lower miR-194 expression levels are associated with unfavorable DFS (*P* = 0.041) and OS (*P* = 0.031). Multivariate analysis further suggests that miR-194 is an independent molecular marker for OS (HR 0.51, 95% CI 0.37–0.71, *P* < 0.001) in particular for lesions ≤ 4 cm (HR 0.43, 95% CI 0.3–0.62, *P* < 0.001). Thus, miR-194 helps to distinguish aggressive small malignancies with worse prognosis from indolent tumors since they should be treated differentially. Expression of miR-126 [9] was found to be increased in ccRCC specimens. Khella et al. found that overexpression of miR-126 was associated with a longer OS in both 481 ccRCC cases (*P* = 0.0009) and 268 patients with larger lesions (>4 cm, *P* = 0.0035) [9]. The involvement of miR-126 in carcinogenesis and progression relied on a number of targets, such as SPRED1, IGF1R, BCL2, CRK, CCNE2, PIK3R2, and several pathways including HIF-1, VEGF, mTOR, and PI3 K–Akt signaling pathways. In comparison to normal renal tissue, mir-210 was upregulated in ccRCC tissue [23]. Patients with high levels of mir-210 had a 1.82-fold increased risk of relapse (HR 1.82, 95% CI 1.11–3.00, *P* = 0.018) and 2.46-fold increased risk of shorter OS (HR 2.46, 95% CI 1.20–5.04, *P* = 0.014). The Kaplan-Meier survival curves showed that patients with upregulated mir-210 had lower DFS (*P* = 0.015) and OS (*P* = 0.011). Similar results for OS were obtained in patients with tumor size > 4 cm. Another miRNA, miR-203, has been found to be involved in carcinogenesis and progression of ccRCC. It is downregulated in* in vitro* cultured RCC cells and ccRCC specimens [24]. miR-203 is an independent prognostic factor of OS for RCC patients (HR 3.071, 95% CI 1.719–6.374, *P* = 0.001), as low miR-203 expression predicts a shorter OS (*P* < 0.05).* In vitro* experiments showed that miR-203 inhibited RCC cell growth and migration, through directly targeting FGF2, as evidenced by partial attenuation of the tumor suppressive effect in a FGF2 overexpression model. Similarly, low levels of miR-497 were a potential independent factor for predicting a short OS in ccRCC patients [25].

A number of miRNAs have been studied for postoperative prediction. The upregulation of miR-630 was independently correlated with lower overall survival rate (HR 3.021, 95% CI 2.074–5.726, *P* = 0.016) in 92 ccRCC patients undergoing nephrectomy [26], and downregulation of miR-217 was linked to poor survival of ccRCC patients [27]. The five-year survival rate of patients with high levels of miR-217 was greater than that of those with low miR-217 expression. By comparing miR-187 expression in postoperative ccRCC specimens and histologically matched normal tissue (T/N), miR-187 expression was downregulated in ccRCC specimens and decreased stepwise with advancing tumor grade and stage [28]. All patients with high levels of miR-187 (T/N > 1) survived 5 years after surgery; in contrast only 42% of those with low-level of miR-187 had survived at this time point (T/N < 0.42), suggesting a suppressive role of miR-187 in ccRCC progression.* In vitro* experiments showed that overexpression of miR-187 inhibited tumor cell growth and decreased motility via directly targeting B7 homolog 3 (B7-H3). Chen et al. reported that miR-129-3p expression was downregulated in ccRCC specimens [29]. Low levels of miR-129-3p were also associated with unfavorable DFS (HR 3.119, 95% CI 1.060–9.175, *P* = 0.039) and OS (HR 3.199, 95% CI 1.075–9.521, *P* = 0.037). 87.5% of ccRCC patients with a miR-129-3p level above the median survived 43 months, in contrast to only 54.2% of patients with a level below the median. The involvement of miR-129-3p in ccRCC metastasis relied on downregulation of a number of metastasis-related genes, including SOX4, p-FAK, MMP2, and MMP-9. Moreover, ccRCC could be discriminated from normal renal tissue according to miR-129-3p levels with an accuracy of 73.5%.

The upregulation of miR-21 has been reported to be correlated with cancer-specific survival of ccRCC patients. The ΔΔCt threshold 1.61 yielded a sensitivity of 66% and specificity of 81% [30]. Downregulation of miR-126 was also associated with cancer-specific survival. A ΔΔCt threshold of 0.57 yielded a sensitivity and specificity of 36% and 100%, respectively. Combination of the two markers achieves a synergistic predictive performance for cancer-specific survival with improved sensitivity and specificity (88% and 75%, resp.). Also, with a combined risk score at cutoff of 6.82 based on the two miRNAs, it predicted 5-year cancer-specific survival rate of 96% for low-risk patients and 48% for high-risk patients.

Another study showed that the ratio of miR-21 to miR-10B (miR 21/10B) was independently correlated with survival (*P* = 0.012) and TNM stage, with better predictive performance than singular miRNA [31]. Analysis of patients without metastasis showed that patients with low miR21/10B had longer disease-specific survival (223 ± 37.1 months) and higher 5-year and 10-year survival rates (84.2%) than those with high miR21/10B, who had a disease-specific survival of 94 ± 63.8 months and 5-year (51.6%) and 10-year (49.1%) survival rate. With the cutoff value set at the median, a Cox proportional hazard regression model indicated that miR21/10B was an independent prognostic factor (95% CI = 1.201–5.736, *P* = 0.016). Thus, miR21/10B could possibly be used for postoperative risk stratification of patients without metastasis.

miR-141-3p and miR-145-5p as posttranscriptional regulators are reported to suppress tumor cell migration and invasion [32] by targeting NRP2 or SLC16A3. Interestingly, simultaneous overexpression of both miRNAs cooperatively inhibits migration by suppressing HS6ST2 and LOX expression, the latter of which was a strong prognostic factor for OS in ccRCC.

Vergho et al. compared ccRCC patients (*n* = 74) and ccRCC patients with a tumor thrombus (TT) extending into the inferior vena cava, which generally indicated a poor prognosis [33]. The results showed that miR-21, miR-126, and miR-221 could independently predict cancer related death in patients with ccRCC. A combined score based on three miRNAs was calculated according to the formula (4.592  ×  ΔCt miR-21) + (−3.892  ×  ΔCt miR-126) + (−1.938  ×  ΔCt miR-221). With a cutoff value of 18.7. The specificity of the risk score for high-risk patients was 90%, while for the low-risk patients it was 87%.

Taken together, specific miRNAs may be potential markers for predicting survival of patients in ccRCC. The combination of multiple miRNAs or miRNAs with clinical parameters, for instance, TNM stage or tumor size, achieved better predictive performance with higher accuracy, sensitivity, and specificity than singular miRNAs. A combined score based on two or more miRNAs is convenient and reliable for risk stratification of patients with ccRCC.

## 5. Prospective

**Figure 1:**
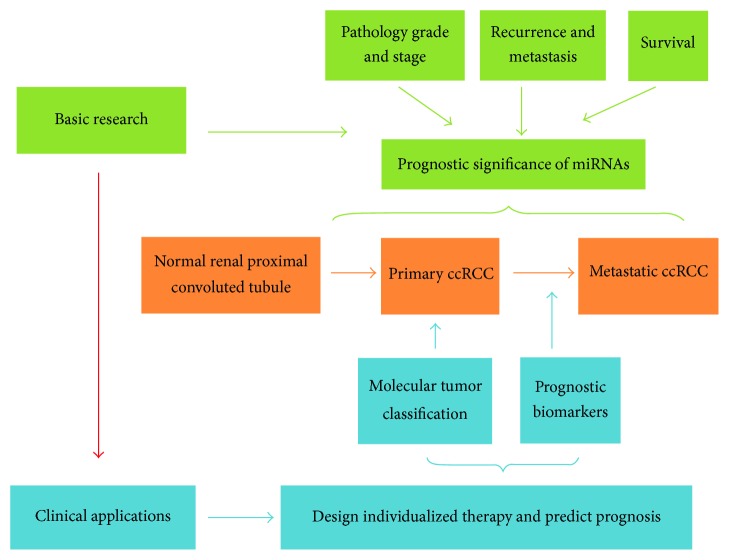
The prognostic significance of miRNAs in ccRCC. miRNAs predict the prognosis of ccRCC in aspects of pathological grade and stage, recurrence and metastasis, and survival.

Accumulating evidence has shown that miRNAs, especially in a group, are sensitive and specific novel noninvasive biomarkers for the prediction of pathological grade, recurrence/metastasis, and survival for ccRCC patients. miRNA may be useful tools for identifying patients at high risk of poor prognosis and thus facilitate personalized therapy and follow-up schedule (Figure 1). Currently, the detection of miRNA relies on quantitative real-time PCR (qRT-PCR). However, interpreting the impact of miRNA expression remains challenging in clinical settings.

Additionally, how to comprehend the mutual and complicated regulation of miRNAs and target genes is another question, since each gene can be affected by different miRNAs, and in turn each miRNA can target multiple genes. Research into the involvement of miRNAs in ccRCC is still at a preliminary stage and requires further investigation before clinical application. And we have summarized all the miRNAs as have been mentioned in our manuscript, some findings (Tables 1 and 3) and functional mechanism (Table 2).

## Figures and Tables

**Table 1:** Studies on miRNAs predicting the prognosis of ccRCC.

miRNA	Ref	Findings
miR-125b	[16]	An independent adverse prognostic factor for the recurrence and survival
miR-124-3	[17]	Its methylation level was associated with the occurrence of distant metastasis, high pathological grade, and increased risk of disease recurrence
miR-30a	[19]	High level predicts longer metastasis-free survival time, an independent predictor of distant metastasis
miR-122 miR-514	[22]	Involve in tumor recurrence after nephrectomy
miR-194	[10]	High level predicts longer DFS and OS, a prognostic factor of OS for tumors ≤ 4 cm
mir-210	[23]	High level predicts shorter DFS and OS for larger tumors (>4 cm)
miR-630	[26]	High level predicts shorter OS
miR-23B/27B cluster	[14]	Low level predicts disease progression and poor survival
miR-29b	[15]	High level predicts advanced TNM stage and short OS
miR-27a-3p	[18]	High level predicts the recurrence of ccRCC in M0 patients
miR-646	[20]	High level predicts longer metastasis-free survival time; and low level predicts ccRCC metastasis
miR-30c	[21]	Involved in metastasis
miR-126	[9]	High level predicts prolonged OS for larger tumors (>4 cm)
miR-203	[24]	Low level predicts poorer OS
miR-217	[27]	Low level predicts poorer survival
miR-187	[28]	Low level predicts advanced tumor grade and stage and poorer 5-year survival
miR-129-3p	[29]	Low level predicts short DFS and OS

ccRCC, clear cell renal cell carcinoma; DFS, disease-free survival; OS, overall survival; TNM, tumor-node-metastasis.

**Table 2:** The miRNAs predicting the prognosis of ccRCC.

miRNA	Changes	Ref	Functional mechanism
miR-23B/27B	Downregulation	[14]	Inhibiting cell proliferation, migration, and invasion
miR-29b	Upregulation	[15]	Promoting apoptosis; inhibiting cell proliferation and invasion by regulating the expression of KIF1B
miR-124-3	ND	[17]	In relation to cyclin D kinase 6
miR-27a-3p	Upregulation	[18]	Inhibiting cell proliferation, migration, and invasion
miR-30a	Downregulation	[19]	Targeting DLL4 and decreasing tumor microvessel density
miR-646	Upregulation	[20]	Inhibiting cell proliferation and cell cycle by targeting NOB1
miR-30c	Upregulation	[21]	Regulating cell motility and adhesion
miR-194	Upregulation	[10]	Involved in ccRCC progression by targeting HIF1A, MDM2, PIK3R2, MAPK1, IGF1R, BCL2, ITGB1, and CRK
miR-126	Upregulation	[9]	Targeting SPRED1, IGF1R, BCL2, CRK, CCNE2, PIK3R2; involved in ccRCC progression through HIF-1, VEGF, mTOR, and PI3K–Akt signaling pathways
mir-210	Upregulation	[23]	Involved in mitochondrial metabolism, stem cell survival, cell cycle regulation, angiogenesis, and cell-cell adhesion
miR-203	Upregulation	[24]	Inhibiting cell proliferation, migration, and invasion by directly targeting FGF2
miR-217	Upregulation	[27]	Inhibiting cell proliferation and migration
miR-187	Upregulation	[28]	Inhibiting cell proliferation, migration, and tumor growth by directly targeting B7-H3
miR-129-3p	Downregulation	[29]	Inhibiting cell migration and invasion by downregulating SOX4, p-FAK, MMP2, and MMP-9
miR-141-3p miR-145-5p	Downregulation	[32]	Inhibiting cell migration by regulating HS6ST2 and LOX

**Table 3:** Studies on combined miRNAs predicting the prognosis of ccRCC.

Sources	Combination	Ref	Findings
Clinical	miR-21 miR-126	[30]	Improving sensitivity and specificity in predicting CSS
	miR-21 miR10b	[31]	The ratio of miR-21/miR10b is associated with tumour nuclear grade, TNM stage, and survival; the ratio is an independent prognostic factor in metastasis-free patients
	miR-21 miR-126 miR-221	[33]	The specificity of CRS for high risk patients is 90%, while for the low-risk patients it is 87%
	miR-125b and TNM stage	[16]	Markedly improving prognostic accuracy

Cell and Clinical	miR-141-3p miR-145-5p	[32]	Cooperative effect on migration and regulation of HS6ST2 and LOX, of which the latter is a strong prognostic factor for OS

ccRCC, clear cell renal cell carcinoma; CSS, cancer-specific survival; CRD, cancer related death; CRS, combined risk score; TT, tumor thrombus; OS, overall survival; DFS, disease-free survival; ND, no data; TNM, tumor-node-metastasis.
